# Management reasoning scripts: Qualitative exploration using simulated physician-patient encounters

**DOI:** 10.1007/s40037-022-00714-y

**Published:** 2022-06-02

**Authors:** David A. Cook, Christopher R. Stephenson, Larry D. Gruppen, Steven J. Durning

**Affiliations:** 1 Office of Applied Scholarship and Education Science, Mayo Clinic College of Medicine and Science, Rochester, MN USA; 2 Division of General Internal Medicine, Mayo Clinic, Rochester, MN USA; 3 Department of Learning Health Sciences, University of Michigan, Ann Arbor, MI USA; 4 Center for Health Professions Education, Uniformed Services University of the Health Sciences, Bethesda, MD USA

**Keywords:** Diagnostic reasoning, Therapeutic reasoning, Clinical decision-making, Diagnostic errors, Education, medical

## Abstract

**Introduction:**

Management reasoning is distinct from diagnostic reasoning and remains incompletely understood. The authors sought to empirically investigate the concept of management scripts.

**Methods:**

In November 2020, 4 investigators each reviewed 10 video clips of simulated outpatient physician-patient encounters, and used a coding form to document observations about management reasoning. The team used constant comparative analysis to integrate empirically-grounded insights with theories related to cognitive scripts and Type 1/Type 2 thinking.

**Results:**

Management scripts are precompiled conceptual knowledge structures that represent and connect management options and clinician tasks in a temporal or logical sequence. Management scripts appear to differ substantially from illness scripts. Management scripts varied in quality (in content, sequence, flexibility, and fluency) and generality. The authors empirically identified six key features (components) of management scripts: the problem (diagnosis); management options; preferences, values, and constraints; education needs; interactions; and encounter flow. The authors propose a heuristic framework describing script activation, selection, instantiation with case-specific details, and application to guide development of the management plan. They further propose that management reasoning reflects iterative, back-and-forth involvement of both Type 1 (non-analytic, effortless) and Type 2 (analytic, effortful) thinking. Type 1 thinking likely influences initial script activation, selection, and initial instantiation. Type 2 increasingly influences subsequent script revisions, as activation, selection, and instantiation become more deliberate (effortful) and more hypothetical (involving mental simulation).

**Discussion:**

Management scripts constitute a key feature of management reasoning, and could represent a new target for training in clinical reasoning (distinct from illness scripts).

**Supplementary Information:**

The online version of this article (10.1007/s40037-022-00714-y) contains supplementary material, which is available to authorized users.

## Introduction

Diagnostic reasoning and clinical reasoning are often viewed as synonymous; yet recent arguments clarify that management reasoning is also part of clinical reasoning, distinct from and possibly more important than diagnostic reasoning [1, 2]. Management reasoning has been defined as: *the cognitive processes by which clinicians integrate clinical information (history, exam findings, and test results), preferences, medical knowledge, and contextual (situational) factors to make decisions about the management of an individual patient, including decisions about treatment, further testing, follow-up visits, and allocation of limited resources *[2]. Whereas research on diagnostic reasoning abounds [3–7], empirical research on management reasoning remains limited [7, 8]. Studies often use treatment decisions as an outcome, but only rarely have they focused on the management reasoning processes that underlie such decisions [9–11]. One group identified [12] and subsequently confirmed [13] 24 clinical reasoning tasks, of which 11 facilitate management. In the absence of further evidence to illuminate the phenomenon of management reasoning, and how it differs from diagnostic reasoning, future research and educational interventions in this field will remain limited.

To empirically explore this topic, we recently completed a qualitative analysis of videos of 10 simulated physician-patient encounters [14]. Grounded in that analysis we identified 12 key features of management reasoning (see e‑Box 1 in the Electronic Supplementary Materials [ESM]). Among these, the concept of the *management script* was entirely new and unexpected. The present paper offers an initial elaboration on this insight.

In that analysis we also planned to empirically examine the cognitive processes (specifically, dual process thinking) that might underlie management reasoning. While ultimately less conclusive than hoped, we share those findings as well.

### Management scripts

Scripts are defined generally as

*high-level, precompiled, conceptual knowledge structures … *[that]* represent general (stereotyped) event sequences, in which the individual events are interconnected by temporal and often also causal or hierarchical relationships; that can be activated as integral wholes in appropriate contexts *[and]* contain variables and slots that can be filled with information present in the actual situation, retrieved from memory, or inferred from the context* [15].

Additionally, *“a script is not a simple list of events but rather a linked causal chain; a script can branch into multiple possible paths that come together at crucial defining points”* [16]. Merging these other theoretical concepts with our empirical observations, we defined management scripts as *“precompiled conceptual knowledge structures that represent and connect management options and clinician tasks in a temporal or logical sequence to facilitate development of a rational management plan”* [14].

The concept of illness scripts dates to 1984 [17], and has been the subject of rich discussion and research [15]. Illness scripts are knowledge structures (mental representations) that reflect how an illness *developed* (i.e., the sequence of events occurring in a given patient) and figure prominently in theories of diagnostic reasoning [18, 19]. Key features of the illness script include the enabling conditions (risk factors), faults (disease pathology), and consequences (symptoms, physical signs, test abnormalities) [17].

By contrast, management scripts are a new concept—namely, mental representations that guide *development* of a management plan. A recent empirical study described “therapy scripts” in the selection of an antimicrobial, but did not extend this model beyond that specific task [20]. Another recent conceptual model of management scripts highlighted their temporal evolution [21], but was not based on empirical data and focused on activities (reasoning, decisions) within the clinician. In our qualitative study [14] we independently developed a model for management scripts that includes activities without the clinician (e.g., involving the patient) as well as within. However, given the multiple features requiring discussion in that report we were unable to fully explore our insights regarding management scripts. In short, previous work suggests that management scripts potentially play an important role in management reasoning, but further exploration of this concept is warranted.

### Dual process thinking

Human memory seems to be divided into working memory (of limited capacity) in which computations are performed and links are established between new and old information; and associative (long-term) memory (of limitless capacity) in which information is stored and retrieved based on patterns (direct association between new information and existing exemplar[s]) [3, 22]. It has long been recognized that human thinking (including clinical reasoning) involves dual processes:

- “Type 1”: intuitive, autonomous, and seemingly low effort (not requiring working memory) and
- “Type 2”: conscious, effortful (requiring working memory), and able to “decouple” supposition from belief (i.e., separation of representations of real-world events from imaginary situations) [23].

Type 1 thinking supports non-analytical clinical reasoning, often manifest in rapid generation of diagnostic hypotheses and pattern matching (association between mental representations [scripts, schemas, propositions, mental models, cognitive maps, chunks] of this case and prior cases) [4].

Type 2 thinking supports analytical clinical reasoning, manifest in slow, deliberate, systematic integration of clinical data, diagnostic possibilities, and additional data gathering (hypothetico-deductive reasoning). It *“enables uniquely human facilities, such as … mental simulation and consequential decision making”* [23]. Research in diagnostic reasoning suggests that novice trainees rely more on analytical reasoning whereas experts typically use more non-analytical reasoning [4].

In our conceptual exposition, we noted that *“[w]e presume that management reasoning reflects a balance of non-analytical processes … and analytical processes …, yet the relative contributions remain unknown. … It seems plausible that management reasoning may be inherently more analytic (deliberate, planned, and systematic) than diagnosis”* [2]. Our rationale was that the numerous factors in play (treatment options, patient preferences, shared decision-making, explicit cost-benefit tradeoffs, etc.) and the absence of a single best solution all combine to both inhibit the generation of needed patterns and prevent their application. However, in the absence of empirical data we suggested further research into this hypothesis.

### Study aims

We aimed to conduct an introductory investigation of management scripts and dual process thinking in management reasoning.

## Methods

We conducted a qualitative analysis of videos of physician-patient encounters, augmented by previously published empirical and theoretical work (listed below). The observational data come from the dataset used in our previous report [14]. The present findings focus on observations regarding management scripts and Type 1/Type 2 thinking, and include previously unreported analyses and interpretations.

### Video selection and coding

We reviewed a convenience sample of 10 videos of staged simulated physician-patient encounters, used in an IRB-approved study of rater training conducted in 2006 [24, 25]. Each video (45 s to 7 min long) portrayed an outpatient follow-up counseling visit between a resident physician and a patient. To enhance the range of observable behaviors, for each medical problem (hypertension, hyperlipidemia, fibromyalgia, diabetes mellitus, and papillary thyroid cancer) we selected 1 poor and 1 superior performance. Six videos (2 pairs) were extemporaneous dialogues between the physician and a standardized patient. Physicians were coached in their level of performance; patients were instructed to respond authentically while portraying the “same” person in both cases. The other 4 videos were scripted dialogues.

All investigators independently reviewed each video at least twice using a coding form (e-Box 2 in ESM) with open-ended prompts, including: *“In what ways was reasoning automatic, fast, and reliant on pattern recognition (reflecting System 1)?”* and *“In what ways was reasoning deliberate, effortful, and slow (reflecting System 2)?”* Each reviewer also documented “epiphanies”—novel insights, themes, and connections that extended beyond what was visible in the video.

### Data analysis and model building

Our analysis used a social constructivism paradigm and constant comparative approach [26], mirroring the method used in a previous study of diagnostic reasoning [13], proceeding in three stages. In Stage 1, author DAC organized each investigator’s raw observations and epiphanies into a 120-page, single-spaced document. In Stage 2, the investigator team reviewed this document individually and as a group, and engaged in multiple voice and electronically mediated conversations to iteratively reorganize, reconceptualize, elaborate, and refine these observations into a new 21-page list of critical insights, of which approximately 20% referred to management scripts or Type 1/Type 2 thinking (see e‑Box 3 in ESM). From this list we identified several distinguishing features (facets of variation) of management scripts.

We recognized that our empirical data were insufficient to deeply probe these concepts, especially since management scripts were an unanticipated finding. Thus, in Stage 3, we integrated conceptual frameworks, theories, and empirical evidence from the extensive literature on scripts, dual process thinking, and diagnostic reasoning (including [3, 4, 6, 7, 15–17, 23, 27]) as we continued to iteratively revise our models (including > 12 h of voice communication and multiple written drafts). We reflected, discussed, and collected new data (i.e., re-reviewing videos) until no additional insights were forthcoming from this dataset. All reviewers came to full consensus on all concepts.

### Reflexivity

Three of us (DAC, SJD, CRS) are practicing internal medicine physicians, and two of us have PhDs in cognitive psychology (LDG) and education/cognition (SJD); these backgrounds surely influenced our perspectives on clinical reasoning and clinician-patient encounters. We also developed this model of management reasoning [1].

## Results

It proved difficult to distinguish “raw” observations from partially or fully developed insights and personal introspections. Thus, rather than quote our own narrative, we present a refined summary of observations and insights (see e‑Box 3 in ESM for additional interim data).

We first outline several empirically derived facets by which scripts varied across videos, then integrate these facets into six key features, and finally describe a heuristic framework for management scripts. Our data did not support strong insights regarding dual process thinking (despite it being a specific aim); we reserve those reflections for the Discussion.

### Management script facets of variation

In nearly all encounters, the physician followed a coherent, fluent, structured sequence of dialogue and activity that appeared to be largely predetermined or preplanned. We interpreted these as outward (observable) manifestations of cognitive scripts (high-level, precompiled mental representations of interconnected events [i.e., clinician tasks]). Scripts guide clinicians as they prioritize and organize management options such as diagnostic tests, treatments, consultations, patient education, shared decision-making, and monitoring to generate a management plan. We discerned that scripts differed in their scope, automaticity, quality, and generality.

#### Script scope

We identified in the video-recorded encounters three distinct script levels of scope. At the most basic, the management *conversation* script constituted a physician-patient dialogue. More broadly, the management *encounter* script encompassed the conversation plus issues such as nonverbal cognitive activity, use of tools (such as the computer), time management, and immediate interactions with other members of the health care team. Broader still, the management *strategy* script encompassed the encounter plus issues such as further diagnostic and treatment options, delayed interactions with the health care team, and monitoring and adjustment of the care plan.

#### Script automaticity

We explicitly sought and documented actions consistent with Type 1 and Type 2 thinking. However, upon reflective analysis of our observations we realized that we could not confidently link observable behaviors with underlying cognitive processes. A slow, seemingly deliberate series of questions could plausibly follow from an effortless, automated decision (i.e., to pursue a “standard” [compiled] set of questions for all patients with this condition [Type 1 thinking]); and conversely a fast, fluent conversation could emerge from a succession of deliberate choices among branching decision-points (Type 2 thinking). Thus, our inferences related to speed and apparent effort/automation seemed likely to denote script quality or communication skill rather than underlying cognitive processes.

Most encounters comprised (at least in part) fast, goal-directed, and seemingly automatic conversations. The clinician quickly focused on a specific (and apparently obvious to them) next step in management, such as initiating a first-line drug, intensifying therapy, or consulting an oncologist. These conversations resulted in highly efficient encounters.

However, we observed many situations in which fast, focused conversations embodied an untailored, simplistic response and were ultimately counterproductive (e.g., a reflexive choice of hydrochlorothiazide in a patient with new hypertension). Such “premature closure” on a treatment option reflected a predetermined, impersonal, inflexible, unidimensional, short-sighted, or excessively vague approach. We imagine that such conversations could arise from a poor script, faulty instantiation, or a knowledge gap. Variations on this theme included:

- Failure to ask about or incorporate patient preferences and logistical constraints
- Making assumptions about preferences (e.g., effectiveness matters more than side effects, cost matters more than effectiveness, long-term health matters more than immediate inconveniences and costs)
- Parroting a plan (e.g., guideline recommendations or other “inherited” script) without understanding why (i.e., without appreciating the underlying nuances of evidence or pathophysiology)
- Conversing without thinking (for example: the patient described in detail that she had implemented recommended lifestyle changes, and immediately thereafter the clinician asked about lifestyle changes)
- Fluency that suppresses discussion (the most egregious example was the extremely fluent monologue in which the patient did not utter a word)
- Simplistic or incomplete management plans (simplistic solutions to complex problems reflect failure to personalize)

Conversely, we observed several encounters in which all or part of the conversation was slow, deliberate, and seemingly effortful. These included asking questions about the patient’s preferences, values, and contextual details (and follow-on questions to probe further details); asking questions to confirm patient’s understanding; identifying limitations or affordances of the health care team and system; and integration of the information thus ascertained. Adjusting the temporal evolution of the encounter to meet patient needs also appeared deliberate. We conceive additional similarly deliberate tasks (not observed in these videos) might include using a decision aid, calculating a personalized risk profile, accessing the electronic medical record, and accessing a computer knowledge resource.

Some of these deliberate conversations appeared planned (part of the mental representation [script]), and some appeared unplanned. Planned events included predictable questions, pauses, and branch points in the conversation. Unplanned events (presumably having no preformed mental representation) included spontaneous patient questions (“interruptions”) and novel situations. In many instances, only after overt action by the patient did the physician slow down, pay attention, deviate from their monologue, or personalize the plan (and once, even following a direct request the physician failed to slow down).

#### Script quality

We observed wide variability in the efficiency and effectiveness of the encounter that seemed to mirror the quality of an underlying script. High-quality scripts pre-empted patient questions, facilitated shared decision-making, and engendered trust and confidence, whereas low-quality scripts were observed in encounters that appeared fragmented, reactive, impersonal, and ultimately dissatisfying for both patients and physicians. We identified four attributes of (observable) script quality, namely script content, sequence, flexibility, and fluency.

Script *content* comprised disease-specific knowledge, and equally important knowledge of local systems and processes (how things actually get done). Robust knowledge enabled detailed and complete descriptions of the disease, options, prognosis, feasibility, and future events.

Some scripts followed a natural, logical temporal *sequence* (e.g., disease description, treatment options, prognosis without and with treatment, use of a decision aid). Others proceeded haphazardly: addressing an issue incompletely before moving on (sometimes returning, other times not), revisiting the same issue repeatedly, or omitting an important detail entirely.

*Flexibility* refers to the physician’s capacity to tailor the management plan (and its development) to the unique patient and context. Adept physicians shaped their narrative from the outset to accommodate what they already knew of patient comorbidities, preferences, and constraints; and paused frequently to ask or answer questions that further directed the conversation to address patient needs. The script thus included not just information to be conveyed, but also awareness of when to pause (e.g., potentially confusing information, or decision points that required patient input) and how the plan might be tailored. We also witnessed flexibility in communication—physicians adapting vocabulary, analogies, examples, and drawings to the individual patient. Less skilled physicians, by contrast, relied on scripts that seemed to be largely predetermined—at least initially, and until disrupted by “unexpected” events or information (e.g., resistance or penetrating questions from the patient).

High-quality scripts were delivered *fluently*—a smooth rhythm that indicated familiarity with the material and efficiently conveyed specific, essential information without repetition or digression. Not only were fluent scripts efficient, but they also engendered comfort and trust (i.e., that the physician had traveled this road before). Less fluent scripts tended to be vague, disjointed, meandering, repetitious, and unnecessarily long. These seemed to undermine confidence, and often left patients with incompletely answered questions and suboptimal management plans. However, a fluent script does not guarantee quality; indeed, one of the least effective encounters was a very fluent “conversation” in which the physician efficiently outlined the rationale for treatment, recommended a single drug, listed side effects, and arranged follow-up, then ended the encounter without allowing the patient to speak.

#### Script generality

We also noted another dimension of scripts, namely the level of generality or abstraction. It seemed that some scripts followed a pattern or framework that could be replicated across content areas. For example, when “initiating treatment for new hypertension,” the physician might proceed with an explanation of the condition, a rationale for treatment, a list of treatment options, and an explanation of the advantages, disadvantages, and costs of specific options. That same general framework could be employed for treating diabetes, hypothyroidism, or stable angina. Other frameworks included “breaking bad news” and “intensifying treatment of a chronic condition”. The best scripts seemed to be those that loosely adhered to a general framework, but had been tailored to both the specific condition and the patient.

### Management script key features

As our understanding evolved, we recognized that management scripts differ substantially from the illness script prevalent in diagnostic reasoning; we summarize these differences in Tab. 1. Most salient, the illness script is predominantly retrospective—it reflects the temporal evolution of the illness up to the point of presentation. Moreover, the illness script is often treated as interchangeable with the diagnosis: multiple scripts (diagnostic hypotheses) are activated early in the case presentation, and these may then be deliberately evaluated to select the script that best matches the available data. In a sense, the illness script is a destination (diagnosis).

**Table 1:** Differences between illness scripts and management scripts

Characteristic	Illness script	Management script
End product	End product is a diagnosis	End product is a management plan
Cognitive representation of end product	Concrete, objective, single correct solution	Abstract, conceptual, multiple correct solutions
Temporal relationship	Retrospective (story [temporal evolution] of patient’s illness up to point of clinician’s involvement)	Prospective (guides temporal evolution of clinician’s future actions)
Script goal	The script is often synonymous with the *diagnosis*	The script is the *pathway* that leads to a management plan
Script activation, selection, and instantiation	Multiple candidate *diagnoses* are activated. The most likely diagnosis (“general clinical picture of disease”) is selected and instantiated (populated with *typical and atypical features* of individual patient’s story)	Multiple candidate *pathways* are activated. The best pathway is selected and instantiated (populated with *known and assumed features* of individual patient’s problem, comorbidities, preferences, etc.). Occasionally, a highly-developed script may lead by default to a specific management plan
Dual process thinking	Often remains largely Type 1 (non-analytical) thinking	Nearly always involves Type 2 (analytical) thinking
Key features	Predisposing conditions; faults; consequences	Problem to be solved; management options; preferences, values, constraints; education needs; interactions; encounter flow (see Tab. 2)
How script is developed/built: Exposure to numerous varied cases enables …	Recognition of atypical presentations of an illness	Tailoring of the pathway (and thereby the subsequent plan) to the unique needs of the individual patient and context
Rate of development	Faster to develop because there are fewer permutations of a single diagnosis	Slower to develop because there are numerous permutations for each problem (varying comorbidities, preferences, interactions, etc.)

By contrast, the management script is decidedly prospective—it guides clinicians as they teach, collect new information, jointly make decisions, and ultimately recommend treatment. Management reasoning involves more than evaluating treatment options against available data to select the best match. It also requires communication and shared decision-making with the patient; dynamic interplay among people, systems, and settings; and ongoing monitoring and adjustment of the plan [2]. As such, we envision the management script as a *pathway* (not a destination) that leads to a management plan (the destination). Our empirical observations suggest six key features (components) of management scripts (Tab. 2):

1. Problem to be solved. Analogous to the “fault” in the illness script, this is usually the diagnosis. It could also reflect other problems such as how to manage hemodialysis while traveling, plans to move to assisted living, or decisions about “do not resuscitate” orders. The problem (diagnosis) can include factual qualifiers, such as disease severity and medical history or comorbidities.
2. *Management options*. These include specific treatments and associated benefits, costs, side effects, and ongoing requirements for monitoring and follow-up. Options also include consultations and further diagnostic tests.
3. *Preferences, values, constraints*. These are non-factual issues that influence management, including preferences and constraints of the patient, providers (clinician and other team members), and system (including time constraints). They can be confirmed, assumed, or at times imposed (e.g., denial of insurance coverage for a desired option, or clinician’s patient workload).
4. *Education needs*. Teaching can occur before decision-making (to explain the problem [what is going on], implications, and prognosis), during decision-making (weighing each option), and after decision-making (to outline next steps).
5. *Interactions*. Interpersonal (human-human) interactions are ubiquitous in management scripts, appearing as key features in our model of management reasoning [1] (i.e., “communication and shared decision-making” and “dynamic interplay among people, systems, settings, and competing priorities”). Human-computer and human-system interactions also figure in many management scripts.
6. *Encounter flow*. This encompasses the timing and sequence of events (teaching, questioning, team conversations, additional diagnostic testing, decision-making, etc.).

**Table 2:** Key features of the management script and example (hypothetical) instantiation

Feature	Potential script elements^a^	Example of an instantiated script^b^
Problem to be solved	– Diagnosis – Patient medical history/context (medical facts [not preferences]): comorbidities, allergies, disease severity, etc. – Level of specificity can vary (“chest pain” or “myocardial ischemia” or “occlusion of the left anterior descending artery”)	– 44-year-old man with newly diagnosed HTN – Comorbidities include obesity and impaired fasting glucose – Allergy to sulfa – Patient *[does | does not]* have edema. Potassium levels are *[normal | high | low]*
Management options	– Drugs – Non-drug treatments – Diagnostic tests – Consultations – Benefits – Costs – Side effects – Monitoring and follow-up	– Hydrochlorothiazide, lisinopril, and amlodipine are top drug options *[(unless edema or abnormal potassium is present)]* – Lifestyle measures including dietary change, exercise, and weight loss are essential – Current lifestyle approach *[is optimal | suggests minor improvements possible | suggests major change required]*
Preferences, values, constraints	– … Of patient – … Of providers – … Of system – … Confirmed, to be confirmed, assumed, or imposed	– Confirmed: Patient understands that HTN is important to treat. Nurse can provide education on diet and blood pressure monitoring – To be confirmed: Patient *[does | does not]* want to try lifestyle measures a little longer before starting drug treatment – Assumed (not typically confirmed): Patient wants to be treated with drug, is willing to take daily drug, can afford drug that costs $ 10 per month, and can return for periodic follow-up – Imposed: Clinician is running behind schedule and feels time pressure that may limit capacity for education and shared decision-making
Education needs	– Before decision-making (what is going on, implications, prognosis) – During decision-making (options) – After decision-making (next steps)	– Before decision-making: what is HTN, what are long-term effects of untreated HTN, what are benefits and costs of prolonged treatment of HTN? – During decision-making: what benefits, costs, and side effects are likely for this particular patient? What can he do to exercise and lose weight? – After decision-making: does patient understand the illness and management plan (i.e., confirmation of understanding), when will he return for follow-up, what questions remain in his mind, does he know how to check his own blood pressure?
Interactions	– Human-human (communication, negotiation, shared decision-making; with patient, nurse) – Human-computer (EHR, knowledge resource) – Human-system (care pathway, insurance preapproval)	– Check EHR to confirm potassium is normal – Invite patient to join in decision making – Patient *[does | does not]* want to use HTN treatment decision aid – Plan to pause and assess understanding at the end – Use computer to send prescription to pharmacy
Encounter flow	– Timing and sequence of events	– Start with teaching about initial explanation of diagnosis, health impact, benefits of treatment – Pause and confirm understanding – Next teach about lifestyle measures – Next describe drug options – Next *[use | skip]* decision aid – Come to agreement on drug (will probably be hydrochlorothiazide) – Pause and confirm understanding – Arrange follow-up with nurse in 2 weeks and with clinician in 2 months – All of this will need to be a bit rushed

*HTN* hypertension, *EHR* electronic health record

^a^List of potential elements is illustrative, not intended to be complete

^b^Underlined text indicates “instantiation” using features of this particular patient and context; text in brackets and italics are empty “slots” that are not yet fully instantiated. The remaining text is relatively generic for all patients who match this (hypothetical) management script. This script reflects the approach of a fairly experienced clinician who sees patients similar to this one every week, and thus can anticipate many of the issues that need to be addressed; less experienced clinicians or clinicians who see this problem infrequently would have less-well-developed scripts. Actions within a given feature (table row) are in approximate sequential order, but the features themselves do not follow exclusively in the order presented herein

### Management script heuristic framework

**Fig. 1:**
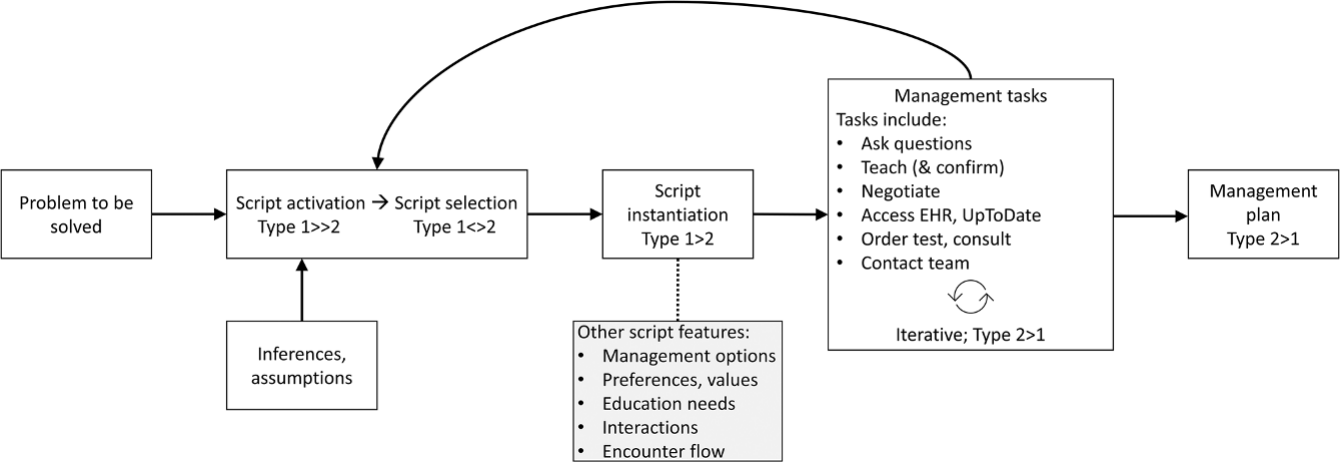
*Model of management reasoning script. *Management scripts are *“precompiled conceptual knowledge structures that represent and connect management options and clinician tasks in a* *temporal or logical sequence to facilitate development of a* *rational management plan”* [14]. Although initial script activation, selection, and instantiation are likely (usually) predominantly Type 1 thinking, with each iteration the script and associated management tasks (usually) employ more Type 2 thinking. The list of management tasks is illustrative, not comprehensive. *EHR* electronic health record

Drawing on the above concepts, we elaborated a tentative heuristic framework for how management scripts might operate in practice (Fig. 1).

Scripts define the temporal evolution of the management plan, linking clinician actions and management options. The clinical problem (usually a diagnosis) triggers the cognitive “*activation*” of multiple candidate scripts relevant to resolving some aspect of that problem. For example, the diagnosis of “new hypertension” might trigger candidate scripts about “hypertension in patient without specific comorbidity”, “hypertension in patient with diabetes”, “low sodium diet”, and “screening for secondary causes of hypertension”. It could also trigger indirectly relevant scripts such as “management of heart failure with preserved ejection fraction”, “chronic renal insufficiency”, and “screening for diabetes, hyperthyroidism, and hyperlipidemia.”.

The number of available scripts and detail of each script will vary. A beginning medical student might have only a general script for “starting a medication”; a primary care physician might have a diagnosis-specific script for “treating new hypertension”; and a hypertension specialist might have separate scripts for patients with diabetes, renal failure, and sleep apnea. As with the illness script [18], management script activation is probably a subconscious cognitive event (Type 1 thinking).

Next comes “*selection*” of the most relevant script—the script most likely to efficiently lead to an effective management plan. We believe, based on research in diagnostic reasoning (illness scripts) [4, 27], that selection involves pattern-matching with prior cases (see e‑Fig. 1) based on various case features (diagnosis, comorbidities, known or assumed preferences, etc.). Script selection probably varies from largely subconscious to largely deliberate (i.e., varying degrees of Type 1 and Type 2 thinking) depending on the familiarity of the clinician with this particular problem and context.

Finally, the selected script is “*instantiated*”. In its general form (prior to instantiation), the script consists of multiple possible branching pathways and multiple empty “slots” [16]—placeholders that can be filled with patient- and context-specific information. Instantiation fills the slots and selects a specific pathway. For the *illness* script, *“[t]he script, or generic event sequence, maps onto the general clinical picture of a disease, whereas each individual patient can be considered an instantiated illness script, with both typical (central) or atypical (peripheral) features, which appear in a certain order”* [15]. For the *management* script, slots might include the severity or urgency of diagnosis, comorbid conditions, current medications, allergies, patient preferences, logistic constraints, etc. Some slots likely remain empty at first, and may be filled later with additional information, or they may remain unfilled. Script instantiation explains how management scripts (and management reasoning) can be tailored to the patient and context. More generic scripts apply across a broader spectrum of cases, but require more effort to fill in empty slots; more specific scripts are faster and less effortful (fewer empty slots), but apply to fewer situations. Experience with a given problem (diagnosis, patient comorbidities and preferences, system constraints, etc.) promotes development of more, and more specific, scripts relevant to that problem; and this in turn promotes greater efficiency in developing a tailored management plan. Tab. 2 provides a hypothetical example of management script instantiation.

Once instantiated, the script guides the clinician in the temporal unfolding of the encounter as the management plan is developed. Cognitively, we propose that the script includes the key features described above (i.e., problem, management options, preferences, education needs, interactions, and encounter; see Tab. 2). Behaviorally, the script guides the choice among various management tasks.

If the encounter proceeds in close alignment with the instantiated script, it may be sufficient to result in an acceptable management plan. However, in many cases the initial script as instantiated will be insufficient. Scripts “do [not] provide the apparatus for handling novel situations” [16], such as an infrequent diagnosis or event, novel connections/insights about the disease/patient/system, or most patients seen by novice clinicians. Moreover, scripts can be thrown off course by distraction (interruption by another script [e.g., realization that labs tests suggest hyperthyroidism]), obstacles (an impeding condition is present or a necessary condition is absent [the patient asks an unexpected question, the preferred drug is too expensive, or a diagnostic test will be delayed]), and errors (an action is completed inappropriately [the clinician fails to address a patient’s strong preference]) [16].

Novel situations, obstacles, and errors all require iterative adjustments. Many adjustments can be made to the initial script by consciously substituting new information or filling previously unfilled script slots (i.e., Type 2 thinking). If more extensive adjustments are needed, or in the case of distraction, new candidate scripts are activated, selected, and instantiated, and the process continues. We observed in some videos a failure to adjust (i.e., a flawed or inadequately tailored script was used); we speculate that reasons for this include limited knowledge or experience (i.e., weak or few scripts) and limited time. It is possible for several scripts to be operating at once [16]; for example, scripts for “newly diagnosed hypertension” and “encouragement of lifestyle change” could run concurrently.

Eventually, the management plan is sufficiently well developed that the plan is implemented, the encounter ends, and the clinician moves on to the next patient. Of course, the management plan continues to evolve over time (the ongoing strategy script) in clinician-patient encounters, between such encounters (e.g., clinician ordering additional tests, or patient deciding to stop taking medications), and through interactions with other members of the healthcare team.

## Discussion

We analyzed videos of simulated physician-patient encounters to better understand how management scripts and dual process thinking operate in management reasoning. We identified differences between illness scripts and management scripts (Tab. 1), explored how management scripts may be selected and instantiated (Tab. 2, e‑Fig. 1), and proposed a model for how management scripts guide the management plan (Fig. 1).

### Integration with prior work

Our conceptualization of the management script differs considerably from the illness script [15, 17] (see Tab. 1), aligning instead with the original construct of the cognitive script *(“a predetermined, stereotyped sequence of actions that define a well-known situation”* [16]). Our conceptualization of the management script also differs from a previous conceptualization in which management script activation leads *directly* to selection of management *options* [21]. By contrast, in our model activation is followed by selection and subsequent instantiation of a script, which guides tasks and temporal events that iteratively consider options (together with other script features) to culminate in a management plan.

### Limitations

This study was intended as an introductory exploration of these topics, and has limitations. First, the videos constituted a convenience sample. They were intended to be realistic, but were limited to 5 conditions in adult outpatient primary care, originally designed for a study of rater training [24, 25], and selected to represent extremes of performances. Four videos were scripted; although this limits their utility in supporting novel insights, this limitation is mitigated as the script writers were not part of our study team. Nonetheless, our findings are best viewed as laying a groundwork for future investigation. Second, our findings are based on observable behaviors. We can speculate about but cannot confirm underlying cognitive processes; indeed, some behaviors could reflect more than one reasoning approach. Third, we authors were both the developers of the guiding conceptual framework [1] and the observers and analyzers in this study, so there is some risk of confirmation bias. Fourth, although our analysis method differed somewhat from typical qualitative analyses, we were transparent in our methods, results and inferences; and our appeal to published literature and theories constitutes a type of data triangulation. We believe our approach was rigorous and sufficiently robust to support the proposed (limited) implications. Finally, many of our insights relied in part on theoretical considerations, and some could have been derived from theory alone. However, the fact that no authors have previously identified these insights (including ourselves, despite extensive discussion spread over several years) highlights the added value of our video-stimulated empirical approach.

### Implications: Integrating management scripts and dual process thinking

Although our empirical observations did not allow direct inferences about Type 1 and Type 2 thinking, these observations stimulated deeper insights that, when merged with existing theories of clinical reasoning, resulted in a novel conceptual model for dual process thinking in management reasoning. Both Type 1 and Type 2 thinking appear to interact with both the mental representation (the script) and the management tasks, usually in an iterative process (e-Fig. 2). Type 1 thinking appears to influence the activation, selection, and initial instantiation of the management script (e-Fig. 1); namely, the initial script is selected quickly, unconsciously, and largely based on pattern recognition. This operates best when the clinician possesses a large library of relevant scripts and each script has a well-developed series of slots, which permits more accurate pattern-matching and greater case-specific tailoring. Tailored (instantiated) scripts serve to focus and simplify the encounter by prompting case-specific questions, limiting options, and guiding education (see Tab. 2). If (when) script revision is required, the processes of activation, selection, and instantiation probably become more deliberate (effortful) and more hypothetical (involving mental simulation)—in other words, increasingly Type 2.

Type 2 thinking takes the script selected and instantiated (presumably through Type 1 processes—at least initially), and deliberately and consciously seeks to align the script with the unique case (to refine the instantiation; and, if needed, revise the script selection). These efforts are helpful if they promote tailoring that culminates in a better-optimized management plan; these efforts are wasteful if they simply consume time and energy (slow and effortful) without improving the final plan. Many Type 2 processes are likely part of the script itself: a largely-automated (Type 1) script could contain planned branch points that briefly pass control to Type 2 (i.e., a back-and-forth between Type 1 and Type 2 thinking) [16]. For example, a 40-second “canned” educational message (Type 1) might pause for a planned question to confirm understanding (Type 2) and then resume. Alternatively, Type 2 thinking likely arises spontaneously when the clinician goes “off script” in response to novel situations, obstacles, and errors [16].

We propose, for illustration, that an airplane’s flight plan is analogous to a management script. The flight plan specifies the destination, route, and anticipated potential challenges. Some plans are detailed, others less so. If all goes as planned, most of the flight is on “autopilot” (Type 1 thinking). Familiarity (repeated experience) with the route, aircraft, and crew and a more detailed flight plan allow more automaticity. Many in-flight adjustments (e.g., response to minor turbulence) are also performed automatically, with greater experience (in general, and with the specific route and aircraft) enabling larger adjustments to be performed with minimal effort. However, deviations from the flight plan are almost always required (Type 2 thinking), especially in long flights during which weather, air traffic, and ground conditions could change dramatically. Thus, the pilot must periodically seek new information, re-evaluate, and redirect. Occasionally, unanticipated challenges or a new destination will cause the original plan to be discarded and replaced. This combination of flight plan (script), autopilot (Type 1), and hands-on adjustments (Type 2) guides actions that ultimately lead to the final destination (customized management plan).

### Further implications for educational practice and research

As an introductory investigation with nontrivial limitations, immediate applications of our findings are constrained. However, we believe that management scripts, and their interrelationship with cognitive processes as discussed above, have potentially profound implications for education research, theory, and practice. We previously speculated that *“[e]xplicit consideration of treatment costs and benefits, use of rubrics to guide management decisions, and thoughtful shared decision-making all suggest a slow, deliberate process”* [14]. If true, this would imply that both training and clinical practice of management reasoning are of necessity rather inefficient. However, management scripts offer a transformative perspective. Rather than teaching only long lists of treatment options, educators might encourage activities designed to promote development of management scripts. Pending further specific evidence, we can borrow methods from what is known about development of cognitive schema and illness scripts [7, 18, 27], including deliberate practice with virtual patients [28], teaching general scripts (such as SPIKES [29] for breaking bad news), learning from errors [30–32], and structured reflection [33, 34].

Our findings suggest avenues for future research. Among these, we need more empirical evidence to test our model, including confirming the distinctions between management scripts and illness scripts (Tab. 1) and reconciling gaps such as how management scripts develop; how activation, selection, and instantiation work; and how Type 1/Type 2 thinking vary over the course of the encounter. Additionally, scripts offer new targets for assessment, including script activation, selection, and instantiation; yet whether and how these processes can be isolated and measured remains to be seen. Future research might again use videos as stimuli (i.e., observable behaviors); such videos might strategically vary according to management script features (Tab. 2) and emphasize the iterative nature of management reasoning (Fig. 1). Lastly, we will need new methods that look beyond observable behaviors to understand deeper cognitive processes.

## Supplementary Information

e‑Fig. 1. Pattern matching in management script activation, selection, and instantiation
e‑Fig. 2. Relationship of type 1/type 2 thinking in management reasoning
e‑Box 1. Key features of management reasoning
e‑Box 2. Management reasoning video coding form
e‑Box 3. “Paper trail” (audit trail): Evolution of insights regarding management scripts and dual process thinking
