# Supplementary material for: Management reasoning scripts: Qualitative exploration using simulated physician-patient encounters
Source: Perspect Med Educ. 2022 Jun 2;11(4):196–206. doi: 10.1007/s40037-022-00714-y (PMC9391545; doi:10.1007/s40037-022-00714-y)
Supplement: Supplementary file 1 — e‑Fig. 1. Pattern matching in management script activation, selection, and instantiation e‑Fig. 2. Relationship of type 1/type 2 thinking in management reasoning e‑Box 1. Key features of management reasoning e‑Box 2. Management reasoning video coding form e‑Box 3. “Paper trail” (audit trail): Evolution of insights regarding management scripts and dual process thinking [file 40037_2022_714_MOESM1_ESM.docx]

Electronic Supplemental Materials for:

Management Reasoning Scripts: Qualitative Exploration Using Simulated Physician-Patient Encounters

Contents

[e-Fig. 1. Pattern matching in management script activation, selection, and instantiation 2](#_Toc97721115)

[e-Fig. 2. Relationship of Type 1/Type 2 thinking in management reasoning 3](#_Toc97721116)

[e-Box 1. Key features of management reasoning 4](#_Toc97721117)

[e-Box 2. Management reasoning video coding form 5](#_Toc97721118)

[e-Box 3. "Paper Trail": Evolution of insights regarding Management Scripts and Dual Process Thinking 6](#_Toc97721119)

[Initial insights about Type 1 and Type 2 thinking and management scripts 6](#_Toc97721120)

[Notes from team meeting 9-17-21 8](#_Toc97721121)

[Additional interim ideas 10](#_Toc97721122)

[Temporal direction 10](#_Toc97721123)

[Script instantiation 10](#_Toc97721124)

[Ideas for Integration of Script + Dual Process 10](#_Toc97721125)

[Important reminders and insights 12](#_Toc97721126)

[Implications For Our Model (Revised Model) 12](#_Toc97721127)

[Team discussion 9-27-21 14](#_Toc97721128)

[Discussion 9-29-21 15](#_Toc97721129)

# e-Fig. 1. Pattern matching in management script activation, selection, and instantiation

Unique case features (e.g., problem [diagnosis], comorbidities, preferences, contextual constraints) are indicated by letters. The cluster of features is compared to cases that have been managed in the past. Several relevant (general, uninstantiated) scripts are unconsciously activated, and the script that corresponds most closely with the current patient's cluster of features is selected (likely a combination of Type 1 [unconscious] and Type 2 [conscious] thinking). This script is then instantiated with case features (including "atypical" feature [B]) together with other details that may be known or assumed. Feature B could be accommodated through subsequent Type 2 thinking. Contrast with Eva's model for diagnostic reasoning (Med. Educ. [2005] 39:98-106).


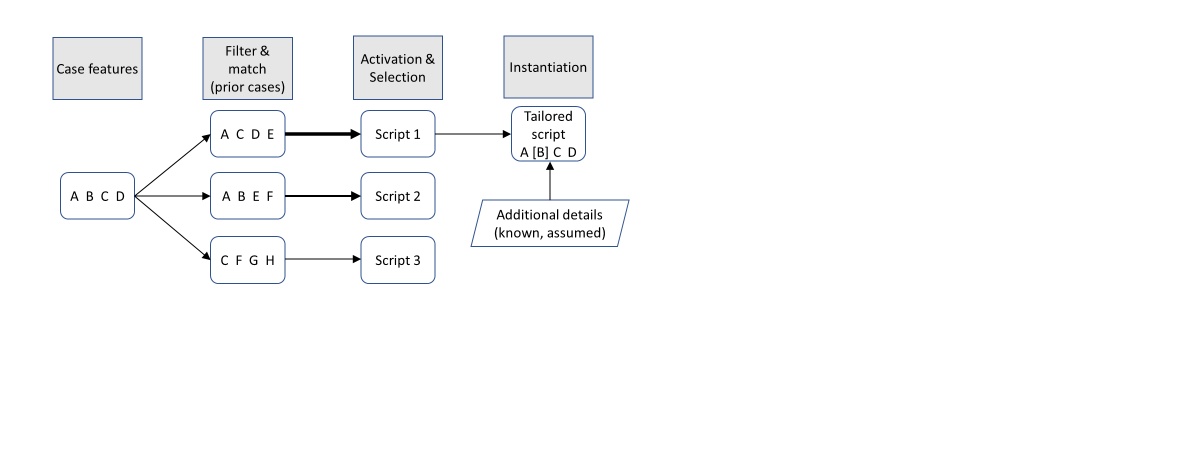


# e-Fig. 2. Relationship of Type 1/Type 2 thinking in management reasoning

Both Type 1 and Type 2 thinking interact with both the mental representation (e.g., the script) and the management tasks, usually in an iterative process. The relative contribution of thinking approach varies depending on the case and the stage of reasoning (likely becoming more reliant on Type 2 in later stages). Contrast with Eva's model for diagnostic reasoning (Med. Educ. [2005] 39:98-106).


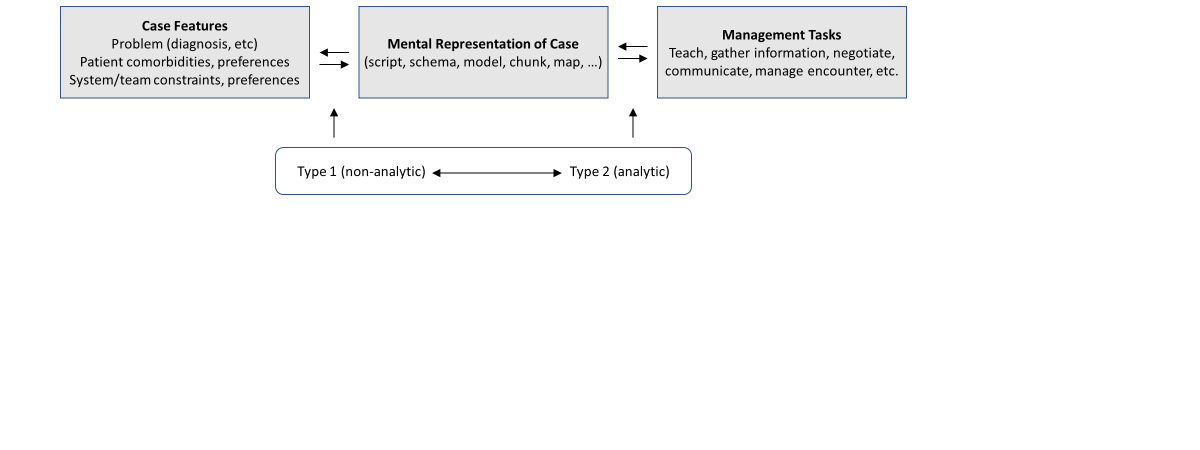


e-Box 1. Key features of management reasoning

Each of these features contrasts substantially from diagnostic reasoning. From Cook et al. (Acad. Med. [2022], in press).

- Contrasting, selection among multiple reasonable / defensible solutions
- Prioritization of patient, provider, and system preferences, values, and constraints
- Communication and shared decision-making
- Ongoing monitoring and adjustment of the management plan
- Dynamic interplay among people, systems, settings, and competing priorities
- Disease-specific knowledge
- Process knowledge (understanding of systems of care – "how things actually work")
- Management scripts
- Roles as teacher and salesperson
- Clinician-patient relationship
- Prognostication (ability to correctly predict future events and share this with the patient)
- Organization (of encounter)

# e-Box 2. Management reasoning video coding form

What features of management reasoning are evident in this encounter (please explain, elaborate)?

Contrasting, selection among multiple reasonable / defensible solutions (no single correct plan)

Prioritization of patient, provider, and system preferences, constraints, and values

Communication and shared decision-making (ascertainment of, integration with patient preferences)

Ongoing monitoring and adjustment of the management plan (plan is a moving target)

Dynamic interplay among people, systems, settings, and competing priorities; uncertain, complex, "situated"

What other features of management or management reasoning are evident in this encounter?

In what ways was reasoning automatic, fast, & reliant on pattern recognition (reflecting System 1)?

In what ways was reasoning deliberate, effortful, and slow (reflecting System 2)?

What unique skills were employed by (or lacking in) this physician to achieve successful management? Are these skills content/context-specific? or would they generalize across clinical problems/settings?

What implications does this encounter hold for assessment of management reasoning (e.g., assessment modalities, domains, temporal evolution)? Include both challenges and solutions.

What feedback on patient management would you offer to this resident (things done well, and suggestions for improvement)? (Consider: if you were caring for this patient yourself, what would you have done differently in managing this patient?)

What was missing from this encounter (e.g., interaction with computer/EHR) that would be important to include in vignettes used in future research of management reasoning?

# e-Box 3. "Paper Trail": Evolution of insights regarding Management Scripts and Dual Process Thinking

## Initial insights about Type 1 and Type 2 thinking and management scripts

Observations documented while reviewing 10 staged simulated physician-patient encounters. Responses have been edited to eliminate redundancy within and between investigators.

**In what ways was reasoning automatic, fast, & reliant on pattern recognition (reflecting System 1)?**

- Pattern recognition
  - Recognize disease 🡪 trigger next step
    - Often seemed to be a knee-jerk response
      - HTN = HCTZ
      - Lipid = Lipitor
      - Cancer = surgeon
    - … how much tailoring needed?
  - ***Premature closure*** on the treatment plan (especially if only 1 option)
  - Some elements ok as closed loop (e.g., first line agents, med side effects) others need personalized (top recommendation)
- Some physicians seemed to repeat the script without a real understanding of WHY … parroting the guidelines or one specific treatment … but no real understanding of nuances of decision foundation or influences
  - How help guide a patient thru a decision that physician does not understand?
  - The depth of navigation limited by depth of understanding, sophistication of script
- Clinical situation (diagnosis? … classification of situation) seemed to trigger a clear pattern
  - Elevated BP 🡪 3 BP classes
  - Elevated A1c 🡪 double metformin
  - Elevated cholesterol 🡪 statin (Zocor)
  - Positive biopsy 🡪 surgery consult
  - … often with little flexibility or depth (next or alternative steps, prognosis)
- Useful as a "flight plan" with key branch points – starting point and general direction for conversation; not helpful if too vague or too locked-in
  - Pause frequently for questions, redirection
- Script
  - Nearly universal!
  - When no script, seemed disjointed, amateur, vague, meandering, uninspiring
  - Errors and gaps in script can have large impact (e.g., omitted option, incorrect effectiveness, wrong or ignored side effects))
    - Must keep up-to-date – evidence and best practices change over time
  - Delivery essential – slow, deliberate script gives appearance of personal
  - Eliminate jargon, include analogies, examples, humor, nonverbal (drawings), etc
  - need to keep script short, focused, accurate, avoid jargon, anticipate/address patient concerns, and allow opportunity for patient to speak/voice prefs and concerns
  - A good script doesn't sound like a script! Sounds natural, personal (and slow!)
  - But can turn into a depersonalized, predetermined plan (caution!)
- Some scripts predetermined, closed, impersonal, unidimensional – ineffective; too vague, too general
  - "Almost like watching a robot read off a recommendation"
  - Fluency (speed / efficiency) not necessarily good …
    - worst video was extremely fluent, but patient not allowed to speak!
  - Predetermined scripts use ASSUMED (default) preferences … like cost, effectiveness,
- "Tailored" script = pre-prioritized for this patient (not an oxymoron)
  - Good if grounded on known preferences, comorbidities, context details
  - Not so good if grounded in assumptions or provider-imposed priorities
- Single focused "next step" is relatively amateur (vs long-range plan) … easy to foresee pattern for very near future (but better than nothing)
- Vary in delivery as well as content
  - Autopilot vs flight plan
  - Example (autopilot, poor): Patient volunteered (report back) about lifestyle, then physician asked about lifestyle (had just been described) … script should have adapted to incorporate this information!
- Robust script
  - Efficient
  - Build confidence: patient says to self "you've been down this road, you know what to expect, I can trust you"
- Simplistic solutions to a complex problem are counterproductive (unrealistic, out of touch).
- Robust script is ESSENTIAL so
  - Can commit cognitive resources to dynamic interplay, adaptation
  - Less system 2 (i.e., robust = more specific, tailored … not cookie-cutter)
  - "Automate what can be automated, so can be more flexible when need flexibility!" … Appearance of effortlessness.
- Generic scripts?
  - Breaking bad news
  - Motivational interviewing
  - Helping to choose among options
    - Equally effective options (e.g., HCTZ, Lisinopril)
    - One option is preferred (in general / for you)
  - Intensifying treatment
  - Is there anything else I can do for you? What questions do you have?

**In what ways was reasoning deliberate, effortful, and slow (reflecting System 2)?**

- Some encounters yes, others no (missed opportunities … failure to probe, elaborate on concerns … too focused on predefined script / destination)
- Useful if helped tailor; not useful if simply slow and effortful!
  - Planned: Key branch points, what information needed (ask questions)
  - Unplanned:
    - Spontaneous – new territory (no precompiled script: infrequent dx/event, novel connection/insight about disease/patient/system, novice practitioner)
    - Patient "interruption" (interruptions should be welcome, but often were not)
- Thoughtful exploration at branch points / interruptions; flexibility, adjust
- Personalization
  - Risk calculator
  - Other personalized risk or recommendations
  - Cost (assumed?? … so is this really personalized?)
  - Anticipate questions
- Repetition of key points
  - Often not productive … sometimes talked in circles (restate same key point, same evidence) – spinning wheels (effortful but unproductive)
- Respond to questions
- Ascertain prefs, values (initial and follow-up questions)
- Integrate prefs, values (rarely explicit)
- Sometimes only showed up when patient forced physician to slow down, pay attention, deviate from script, or personalize plan/messaging
- Adjust encounter to meet patient needs
  - Re-summarize all data
  - Ask additional questions to confirm diagnosis, etc

## Notes from team meeting 9-17-21

1. Is all negotiation automatically Type 2? Or can it remain Type 1 (pattern-matching) if I am following a well-instantiated script without deviation?
   1. Brains are wired to simplify the world … we want to chunk, and turn Type 2 into Type 1. There will inevitably be Type 1 "patterns" with any action that is repeated.
   2. Management reas involves preferences and negotiation and thus is more Type 2, because a label does not require negotiation.
   3. But it is NOT purely anything … it is incredibly dynamic, evolving, back-and-forth.
   4. *** What about script instantiation? …
2. Reaction to Fig. 1 (early draft)
   1. Where does Negotiation fit in?
   2. Chris asks: are there multiple levels of chunking (fixed conditions, preferences separately) and then we pattern match on these chunks.
      1. Cannot really tell that this is what is really happening … but it seems to fit
      2. Idea is to simplify the universe! My initial model is way too complex
      3. Chunking is more likely and fits with what we know …
      4. Of course, we need empiric evidence
   3. This is too uni-directional … real reasoning is very bidirectional, not static
      1. Way more complicated!
   4. Management is a definition of the situation – evolving definition (priorities, etc)
   5. How many scripts would be instantiated?
      1. Larry says multiple scripts instantiated … constantly shifting scripts as pattern evolves … bidirectional (preliminary script guides information collection that guides next layer in script)
      2. Instantiation is more than object recognition … we fill in details (missing information) in Type 1 (e.g., image perception, case missing details)
      3. Not linear – go back for more information
      4. Fill in missing details is important
   6. We DO have multiple script "hypotheses" (potential plans) that are filtered and prioritized … as we work thru these hypotheses the number of candidate scripts grows and shrinks
      1. Distinction of activation vs selection
         1. Activation is unidirectional (will pop up as a candidate script at any time)
         2. Selection is more conscious, deliberate … could still be pattern recognition, but often and more Type 2
      2. Evolves throughout the entire process
      3. Work thru it as we go … highly active, dynamic
   7. ASSEMBLY of multiple mini-scripts (chunking)
      1. Discrete aspects of management plans … not entirely unique pattern for the entire plan
      2. Our brain doesn't does not do mega-plans … we chunk
      3. What drug … what FU … what education … dealing with disagreement … etc.
   8. What LEVEL are we thinking?
      1. Initial activation?
         1. These are largely pattern
         2. Multiple (more than just communication) …
         3. Linked by acquisition and assimilation of additional information (often negotiation)?? … a blend of Type 1 / 2
      2. Encounter?
         1. Negotiation is ongoing, bidirectional
      3. Strategy?
      4. Progressively Type 1 🡪 Type 2 with higher levels
   9. Time (surrogate) and effort (sina qui non) = Type 2
      1. Mini-scripts can be compiled (T1) or deliberate (T2) depending on expertise / experience
3. Why do we care about Type 1 vs 2?
   1. Brain automatically moves us to Type 1 …
      1. Fast, efficient; allows us to dedicate cognitive resources to other activities
      2. We want to create fast, efficient learners = need to help them develop the right chunks
         1. What are the core mini-scripts?
         2. How can we get explicit about development?
         3. How to teach to develop better patterns, better scripts? … How develop more efficiently? … Deliberate!
         4. Move beyond content … "How do you actually DO this?"
4. Other questions
   1. How does illness script intersect (hand over?) to management script?
   2. How does pattern matching play a role (e.g., how much does illness script influence the management pattern)? … Is this bidirectional?

## Additional interim ideas

### Temporal direction

- Illness script is historical … the ***development*** of the illness, and what brought patient to the clinician.
- Management script is prospective, evolving … the future (current) development of a plan; dynamic, ongoing.

### Script instantiation

1. For illness script: "The script, or generic event sequence, **maps onto the general clinical picture of a disease**, whereas each individual patient can be considered an **instantiated illness script**, with both typical (central) or atypical (peripheral) features, which appear in a certain order."
   1. **How would general vs instantiated manifest in management script?**
   2. We have touched on this briefly above … might be good to elaborate on this point (and perhaps scale back the "quality" features).
   3. I think the issue of "generality" is distinct from "general script" … it is more a meta-script or framework (not disease-specific).

### Ideas for Integration of Script + Dual Process

- Use T2 to select the script ("**I've got a script for that!**")
- Interaction itself is **high cognitive** demand (multiple concurrent tasks!)
  - **How lower the cognitive load**?!?!
- Must ALWAYS narrow the list of options – always more than could possibly be explored … how do this? (script, type 1?)
- Analogy of flight plan (general script, Type 1 process) vs response to complicating issues (script instantiation, Type 2 process) … see above
- Organization (sequencing) of **encounter** can be unconscious (passive ??T1) or conscious (deliberate, T2).
- One overarching question: are illness scripts commonly viewed as Type 1? … these management scripts were not! … A couple were Type 1 knee-jerk; but most were a blend of T1 and T2 … is this a **distinguishing feature** of management script? Should we call it out?
  - LG says " The original script idea pre-dated the Type 1 vs 2 distinction and was not very clear on conscious or unconscious use. My understanding of the script is that it can be both 'intuitive' (Type 1) and explicit (Type 2)"
  - CS says " I agree with the above. Type 1 seems to be more pattern recognition intuitive (For example, you have diabetes, we need to treat it, here's your mediation.) This would parallel with inflexible scripts. … Type 2 is more thoughtful and reflective (for example, you have diabetes, it needs to be treated, here are our options and why I think this treatment is best)."
- Is every case "complex"? Or are some cases simple, able to be resolved purely (or largely) using Type 1 thinking and a well-developed script?
- Tasks vs reasoning
  - Most of these codes deal with management tasks; not a lot here about the underlying reasoning (i.e., what's going on inside the physician's brain).
  - What is going on inside that black box? How do they actually DO the integration? How do they DECIDE upon a specific course of action? …
- Single person vs "**collective cognition**"
  - How much mgt reasoning occurs in the physician's brain, how much resides with the patient, and how much takes place in the "space" between physician and patient?
  - Is it the physician who "reasons"? Or is the "reasoning" itself a negotiation?

### Important reminders and insights

1. Illness script is past-focused (how illness developed); management script is future focused (what will we do now? "live" unfolding of events)
2. We are confusing what we can observe (= conversation and encounter management) with the actual script (which is deeper) ... the observable behaviors reflect, but are NOT synonymous with, the deeper actual script.
   1. Much of what we are calling "script" is only the outward (observable) manifestations of psychological or cognitive scripts (high-level, precompiled knowledge representations of interconnected events).
   2. Conversation, encounter, and strategy are NOT different levels of script … Conversation script is not psychological scripts at all; it is closer to a movie script – a predefined series of words. Many of the encounter "scripts" were also largely predefined.
   3. I DO think we should describe what we saw! (these are our empiric data) ... But then we need to go deeper and clarify the real script
   4. The real script has 5 (or maybe 6? see Word doc) key features
3. There is only 1 management script (not a whole bunch of mini-scripts) ... the mini-scripts or sub-scripts are just tasks and options that constitute part of the larger management script.
   1. I think we nailed it in our definition (manuscript submitted) as "Precompiled conceptual knowledge structures that represent and connect management options and clinician tasks in a temporal or logical sequence to facilitate development of a rational management plan"

### Implications For Our Model (Revised Model)

1. We observed a "coherent, fluent, structured sequence of dialogue and activity that was largely predetermined or preplanned" … but this was an OUTWARD MANIFESTATION of the script (not the script itself).
2. There are NOT multiple levels or layers of scripts … there is 1 "management script."
   1. What we previously called "levels" are really just outwardly visible tasks within the script
      1. Conversation = visible
      2. Encounter management = visible
   2. The "quality" features (content, sequence, flexibility, and fluency) are really features of the visible manifestation – not the script itself! (I think we can still talk about these, but need to be more nuanced …)
3. Key features should FOCUS ON (or support)
   1. Commonalities (abstraction of information)
   2. Principles (fundamental laws, etc [would include EBM, guideline adherence, etc])
   3. Feature consolidation (the way information/data is transformed, aggregated to create meaning)
4. The key features are … (listed below in main text)
   1. *** **Need to confirm** *** that these truly help with the points raised
      1. #3 above (commonalities, principles, features) and
      2. #5 above (similar problem = similar solution, focus on distinguishing features, meta-cog encounter mgt)
      3. … and if they do not, then perhaps consider revising this list
5. Other points to ADD to this discussion
   1. Strong vs weak scripts
      1. Generality (general approach) … as discussed
      2. Highlight/clarify that general approaches (weak) can form foundation of a strong script (deliberate integration of the general model)
   2. How develop scripts? … Speculation beyond empiric data, but important to mention (in Results [I think ok to do this if we add "integration with prior conceptual models arising from diagnostic reasoning" to methods]? Or in Discussion)
      1. Accumulation of exemplars (spontaneous) 🡪 abstraction
      2. + integration with general model? (deliberate, strategic)

## Team discussion 9-27-21

- Steve notes that there are developmental aspects + nuanced aspects of the problem that make it incredibly complex, more dynamic
  - Restaurant script:
    - 5 year old has naïve script
    - If someone comes in and says they need help jump-starting their car – creates need for a new script
  - Not a simple diagnostic label … patient with multiple needs … pulling from multiple scripts that are assembled into a script for this patient
  - Cannot set aside script development … we have various scripts at widely disparate levels of development (some things we are very comfortable, others are new)
  - Our mental constructs must merge to meet needs of individual patient … problem gets very nuanced … where does mgt script live – is it connected with diagnosis?
    - How are they put together (assembled) in the moment? … When problems are interrelated … are there pieces that are assembled?
    - Not all stored in 1 mental block
    - Not a static diagnosis 🡪 1 script will not work (unless something we see over and over, and have really developed a true script … eg cardiologist)
  - Not a single diagnostic label … management is a very complex, nuanced
  - Where does this live? Is it linked with the illness script? How is it activated?
- This is a lot of conjecture and hypothesis.
- Larry: this is moving way beyond what we know
  - Original script was computer science, focused on sequence of events
    - Slots and temporal evolution
    - Never there in 1 unit – once time / event passed, cannot go back, script/behavior goes on … very environment-dependent … guiding behavior in an environment
  - Feltovich moved in very new direction … generic representation … schema, prototype
  - If we go back to original, it makes sense … e.g., when talking with patient about lipids & statins … temporal evolution, slots, etc
  - Larry says we are making the script too broad, all-encompassing; too much content
    - Restaurant script is simple … like computer program
      - Need to sit down
      - Need to order food
      - Need to get drinks
      - Need to eat
      - Need to pay
  - Larry likes the focus on tasks/activities, sequential events
    - Says script different from schema
    - Focus on series of events … script does not have enduring psychological presence like a schema – does not continue to "exist" after the series of events are complete
- This is a lovely self-analysis and integration with theory!
  - We have TONS of questions! (See Steve's comments above)
- Larry suggests: shift from theory model to "heuristic framework"
  - All theories are wrong, some are useful
  - Help frame future questions and research
  - Larry says important questions
    - Role of time
    - "Corporate" nature of management
    - Systems lens: clinician is a cog in the wheel
- Steve
  - Is 1 script instantiated? Or multiple scripts?
    - Activation = multiple options
    - Selection … pattern match + type 2 dialogue
    - 🡪 Need to distinguish activation + selection
    - Instantiation (for Dave) is population of script
    - Steve says selection involves patient conversation
      - Dave is not sure … I think selection is internal
  - We have blended lots of ideas … many authors (in and out of medicine)
  - Not only negotiation; also that the Outcome is derived from the patient itself … I don't know what "success" is until I've reached the end of the conversation! Too nebulous – both the **destination and the journey/path are undefined**!
  - What's been instantiated will likely be shaped during the patient conversation, and may be permanently impacted
- Larry
  - Wrestling with illness script (diagnosis) vs management script
  - Illness (diagnosis) is cut and dried
  - Management … so much more complex in the encounter … every patient is different, never a cookie cutter response … always "It depends"
  - Vocab/definitions
    - Activation is internal Type 1
    - Selection is more Type 2
    - Instantiation is populating the slots/details

## Discussion 9-29-21

- Increasing clarity that illness script and management script are very different. Discussed script activation, selection, instantiation; for illness, selection is often deliberate (information collection … although not always [e.g., written clinical case]), whereas for management it is usually tightly linked with activation.
- Repeatedly used the flight plan analogy (take off from MSP, fly to ATL … widely varying degrees of deviation from original plan [routineness]) … This is a really useful analogy – must retain!
